# Mr. Foge, on Delivery of the Placenta

**Published:** 1804-01-01

**Authors:** A. Foge

**Affiliations:** Newcastle


### *To the Editors of the Medical and Physical Journal.*

GENTLEMEN,

YOUR correspondent, Mr. Marson, in a late Journal, when he attempts to deliver the placenta, says, he applies his left hand to the sacrum, and the right to the parietes of the abdomen, on the region of the dilated uterus; makes a slight pressure with both hands, and increases it gradually, till he exerts what he calls *sufficient* force for two minutes. He frequently relieves his right hand, with the idea of forming a *kneading* motion. He then ties and cuts the navel string, and says, "I again apply to the funis, and find find the placenta waiting for me, either at the os externum, in the vagina, or so as to be brought away directly, or in a minute or two afterwards; if it should not, I apply pressure as before, and the business is generally compleated; but I should repeat it again and again if necessary." He does not say that he ever applied to the funis till he had performed the operation of kneading nearly two minutes, tied and cut the funis; and the placenta was generally waiting for him, and ready to be brought away in a minute or two afterwards.

Whatever use the right hand was of, in forwarding the separation of the placenta, I see no use the left was of when applied to the sacrum. Does Mr. M. apply such force with the right as to move the woman on the bed, and is obliged to keep her steady with the left? He certainly applies more than a slight pressure. In my humble opinion, it would be more to the purpose, to apply both hands to the parietes of the abdomen, and press with them alternately in the true stile of kneading.

As the operation of kneading has been no part of my practice, I can say nothing in its favour; but I seldom meet with a case in which the placenta is not delivered in the same space of time, *i. e.* in four minutes or a little more, without the operation of kneading. I have attended too many labours, where, from the long continued strong pains the parietes of the abdomen were so painful when touched, that they could very ill bear the necessary pressure of a bandage, or even to allow the bed-clothes to press upon them. There, if the placenta did not advance readily, the kneading process would have been a cruel operation, especially if repeated again and again.

Among other methods, Dr. Denman recommends "making a *moderate* pressure with the expanded hand upon the abdomen, to aid the action of the uterus; but says, the term moderate has no precise meaning:" It is not so precise a meaning as Mr. M's sufficient. He afterwards recommends, "the application of the half closed hands to the abdomen." I suppose he means the hard knuckles. This looks like kneading in earnest. I am very well assured that if the knuckles are applied with such force, as to separate the placenta, the woman must suffer more pain, and the parts will be more injured, than by the introduction of the hand, which might safely and effectually perform the separation and exclusion, which no man, I think, will pretend to promise by the operation of kneading. I have removed several soon after the birth of the child, when I discovered an an irregular contraction, and even on account of the atonic state of the uterus, and also when there was an unusual firm adhesion. And, I speak from many years experience, I have not had cause to repent having done so. I have never seen injury nor inconvenience in consequence of acting in that manner; but have heard of many unfortunate cases, where the placenta was left to nature, or allowed to remain too long before it was extracted by art.

Dr. Denman tells us, "Though the placenta may be retained for many hours after the birth of the child, if we be convinced of some degree of descent, especially if we can feel that part of it into which the funis is inserted, we have no reason to be alarmed, or to hurry its exclusion, unless there be an *existing* hæmorrhage; then the placenta may be suffered to remain till it is excluded by the action of the uterus; or, as it descends, the most gentle assistance may be given by pulling at the funis to extract it, without any apprehension of danger, whether it be detained two, or even twenty-four hours, because we have at all times under these circumstances, an easy and certain command of it."

I beg leave to remark on this paragraph, that the word many, like the word moderate, conveys no information; it may mean 3, 36, or 360 hours. Dr. Denman says, "I once saw an instance of a whole placenta retained till the fifteenth day after the birth of the child, and then expelled with little signs of putrefaction, except upon the membranes; the whole surface which had adhered exhibiting marks of fresh separtion. The recovery of this patient was very fortunate, for I have seen several other cases of a similar kind terminate fatally." So much for leaving the placenta to be expelled by the action of the uterus. He says, if we are convinced of the descent, &c. we have no reason to be alarmed, unless there be an *existing* hæmorrhage. He does not tell us how far he introduced a finger to feel the part where the funis was inserted. If that part was descended to the os externum, I think it bad practice to allow it to remain till it is excluded by the action of the uterus, "when we have an easy and certain command of it."

I will also take the liberty of remarking on what Dr. D. calls an *existing* hæmorrhage. I have been called to attend several women, on account of dangerous faintings, who were suffering from *existing* hæmorrhage; where the midwife, instead of attending to the woman, had chose to attend to the dressing the child, and had left the pla-

 centa centa to be excluded by the action of the uterus. I there found, in consequence of the placenta being left, there was no alarm about hæmorrhage, as the vagina was blocked up by the placenta; and, from the atony of the uterus, and the want of a pressure, the uterus was almost as large as before the birth of the child, being filled with blood; from which it is evident, there may be an *existing* though not *apparent* hæmorrhage.

No man can tell whether the placenta, if retained two hours, will be excluded in other two, or in twenty-four hours; nor can he tell whether it will be excluded at last, without art or force, unless "he has an easy and certain command of it." If that is granted, I am certain the hand may be introduced with incomparably more ease soon after the birth of the child, than it can be after twenty-four or forty-eight hours, when, particularly in laborious or preternatural labours, the parts are in a state of inflammation, and much contracted, as I have too often experienced. I also feel for the woman and her friends; her and their anxiety is very great, till there is what they call a happy separation. And as I see no good reason for delaying the extraction so long, I think it safer and better to extract it when it can be done so easily; and think it a good general rule, according to Dr. Denman, "to prefer the introduction of the hand to separate and bring the placenta away, rather than incur the danger of separating the funis from the placenta, or of inverting the uterus by pulling the funis."

Mr. Marson says, "Admitting the placenta to be urged forward by pressure, it is very clear that the hour-glass contraction, as it is called, when this is practiced, cannot take place." Allowing his argument full force, instead of begging the question, I have to observe, that in every hour-glass contraction I have met with, the contraction succeeded the birth of the child so quickly that there was no time to apply pressure; and, sanguine as he is about his method of kneading, I am afraid, if ever he meets with such a case, he will find his pressure inadequate to force the placenta through the contraction. He seems to challenge such a case; I wish him success.

Dr. Denman says, "This assistance," meaning the pressure with the half closed hand, "cannot be given in the worst cases, that is, when the uterus is not at all contracted, or contracted irregularly." If that is the case, this new method of kneading can seldom or never be requisite, as, if the uterus contracts regularly, and in proper time, the placenta will be excluded; with very little assistance, in the same space of time as those under Mr. Marson's management, as observed in the beginning of this paper, viz. four minutes, or a little more.

In a former number of the Medical and Physical Journal, I observed a case on the delivery of the placenta, by Mr. Bartley. He says, the woman was delivered without assistance before he arrived, that considerable hæmorrhage had ensued, but had ceased. The woman was extremely weak and exhausted by the effusion, &c. On introducing his hand, he found a *morbid* adhesion had taken place near the fundus uteri. I have never considered an adhesion, though firmer than what are generally met with, as deserving the name of *morbid*. It appeared from the hæmorrhage which succeeded the birth of the child, that the whole of the placenta had not been in a state of morbid adhesion. He waited four hours and gently strained the cord during the pains. The woman became very importunate, to have the placenta taken away at all events. He again introduced his hand, and removed several large clots of blood which obstructed its passage. Then cautiously introducing his fingers round the whole substance of the placenta, gradually detached it, and brought it away entire. Slight hæmorrhage and repeated faintings alarmed him, and made him repent that he had not persisted in his first resolution of trusting to nature for the exclusion of the placenta. If Mr. B. had removed the placenta the first time he introduced his hand, there might have been the same slight hæmorrhage and faintings as took place at last, and no more. But from whence originated the large clots of blood which obstructed his hand? They were formed during the four hours he sat pulling the cord; and which was a very effectual method of occasioning a return of the hæmorrhage which had appeared at first. But as the blood coagulated in the uterus, he did not suspect any hæmorrhage *existing*. By his delay he gained no advantage, but the woman lost all the blood which formed the clots, which might amount to one or two pounds. No wonder she had repeated faintings. The woman could not be stronger at last than she was at first, when he introduced his hand, after losing so much more blood. His account of separating the placenta at last, does not make it appear that there was any morbid adhesion. We are told of schirrous and cartilaginous adhesions, but this did not appear to be one of these. The placenta remained *partly* attached to the uterus, probably from its atonic

 state state induced by the primary hæmorrhage. Upon the whole, the woman's importunity, possibly, contributed to save her life.

Mr. B. concludes with observing, "that the placenta may be retained with safety, a much longer time than is generally admitted." The above case, in my opinion, does not support that position. I am one of those, who can see no safety in allowing the placenta to remain four hours under the forementioned circumstances, I mean an *existing* though not apparent hæmorrhage, attended with great weakness and faintings.

Mr. Marson drops some hints on the use of pressure in flooding cases. When properly applied, it is the only remedy to be depended upon. I have often wondered that it is never mentioned in Systems of Midwifery. Nothing but cold, wet, icy applications, which as I have always condemned I have never used, nor have I ever lost a patient in flooding. Mr. Marson also hints that he may illustrate and enforce the application at some future period. I for one of your readers wish much to hear his sentiments on the subject, when perhaps I may be induced to give mine.

*Newcastle,* I am, &c.

*December* 1, 1803. A. FOGE.

